# Molecular analysis and validation of primitive races peach palm (**Bactris gasipaes**) by means of markers RAPD

**DOI:** 10.1186/1753-6561-8-S4-P124

**Published:** 2014-10-01

**Authors:** Cirlande Cabral da Silva, Doriane Picanço Rodrigues, Spartaco Astolfi Filho, Charles Roland Clement

**Affiliations:** 1 Instituto Federal do Amazonas (IFAM), Manaus, Brazil; 2 Universidade Federal do Amazonas (UFAM), Manaus, Brazil; 3 Instituto Nacional de Pesquisas da Amazônia (INPA), Manaus, Brazil

## 

Molecular markers were used to examine the genetic variability of eight landraces of peach palm (*Bactris gasipaes *var. *gasipaes*) and two wild populations (*Bactris gasipaes *var. *chichagui*), their relationships and genetic structures. Two hundred plants of these 8 races were used and 18 plants of the two wild populations, one from the Magdalena River, Colômbia, and the other from the Xingu River, Pará, Brazil. Eight primers were used to generate RAPD markers, of which 124 markers with 101 polymmorphic. The observed heterozygosity was 0.38, with 93% polymorphism, both slightly greater than in earlier studies. The "gene flow medium" was 1.12 among the races of peach palm, which is explained by the distance between most breeds analyzed. These values are significantly larger than those of [1], because it is a less accurate marker for this type of analysis. The Amazonian landraces had greater heterozygosity and % polymorphism than the Central American race. The structure of the dendrogram with the four previously studied landraces, essentially validating it and confirming two landrace groups - one Occidental and one Oriental. When the Juruá landrace was added to the analysis, it joined the other Occidental races, as expected by its geographic position. When the other landraces were added to the analysis, a consistency and some problems were observed. The consistency was that all the landraces joined the Occidental group, expected by their geographic positions. The problems were the grouping of the Vaupés landrace with the Juruá landrace, which are geographically distant and have different fruit sizes and shapes. The relationship between the Cauca landrace and the Inirida landrace was also problematic, since they are geographically separated. The two wild populations joined the landraces at a great distance, suggesting that they did not participate in the domestication of the cultivated landraces and indirectly reenforcing the hypothesis of a single origin in southwestern Amazonia.
